# Transrectal high-intensity focused ultrasound for the treatment of prostate cancer: Past, present, and future

**DOI:** 10.4103/0970-1591.60436

**Published:** 2010

**Authors:** Luigi Mearini, Massimo Porena

**Affiliations:** Department of Urology, University of Perugia, Italy

**Keywords:** High intensity focused ultrasound, prostate cancer, indications, outcomes

## Abstract

Upon a review of recently published articles on high-intensity focused ultrasound (HIFU) in the treatment of prostate cancer, we evaluated the current status of HIFU as a primary treatment option for localized prostate cancer and its use as salvage therapy when radiation failed. We also briefly discuss current issues in indications, definition of response, and finally the future of HIFU development.

## INTRODUCTION

Prostate cancer is with higher incidence in men,[1] and is the second leading cause of cancer mortality in industrialized countries. This high incidence has resulted in an increased number of patients requiring therapy. Widespread use of prostate-specific antigen (PSA) testing and the continuous efforts for early detection of early prostate cancer had increased the number of patients with localized prostate cancer, suitable to a curative approach.

The current management of localized prostate cancer could vary from expectant management (for men with low risk disease and a life-expectancy <10 years) to radical prostatectomy or radiation therapy with external beam radiotherapy (EBRT) in patients with a long life-expectancy, which represents gold standard of therapy in young men with localized prostate cancer.

Aim of cure is to enhance “quantity” of life, even if the increasing emphasis on achieving the best survival benefit while better preserving “quality of life” (in terms of side-effects, continence, and potency), the so called TRIFECTA, especially for the population aging and the incidence of low-grade prostate cancer, has led to the increased popularity of minimally invasive treatment, with the emergence of new nonsurgical therapeutic options for localized prostate cancer.

This refers to the use of a wide range of techniques for local target ablation of the prostate gland with minimal damage to the surrounding tissue. Three-dimensional conformal radiotherapy, brachytherapy, intensity-modulated external beam radiotherapy, and laparoscopy had gained acceptance in the treatment of localized prostate cancer; other experimental technologies such as photodynamic therapy, and microwave and radiofrequency interstitial tumor ablation are currently under investigation in early clinical trials.

Transrectal high-intensity focused ultrasound (HIFU) is a relatively new technology,[2] which is capable of inducing instantaneous and irreversible coagulative necrosis in all biologic tissue by thermal effect (absorption of ultrasound energy converted into heat) and cavitation. The focused ultrasound waves are emitted from a transducer and absorbed in the target area, with limited damage to the surrounding tissue, and appears to be a very attractive therapy.

Radical prostatectomy has long been regarded as the gold standard form of therapy in patients with organ-confined prostate cancer. Despite excellent long-term survival rates, surgery is associated with significant morbidity; in addition, surgery is not indicated for patients whose life-expectancy is <10 years.

HIFU is a noninvasive technique for the thermal ablation of tissue. Together with brachytherapy,[3] cryosurgical ablation of the prostate,[4] three-dimensional conformal radiotherapy, and intensity-modulated external beam radiotherapy,[5] it is one of the most attractive options for the noninvasive treatment of localized prostate cancer in patients with a life-expectancy of less than ten years but with a significant tumor or who do not accept the considerable morbidity associated with radical prostatectomy.

In particular, HIFU is well-suited for the anatomic position of the prostate gland, easily reached by transrectal approach.

## BACKGROUND

Ultrasound is a vibration, a wave with a frequency not audible by human ears; it is produced by a crystal or transducer. The ultrasonic wave deposits an amount of energy in a tissue, with the production of a thermal damage. In clinical and diagnostic practice, this thermal injury is limited.

HIFU is an acronym for high-intensity focused ultrasound: In this way, wave intensity if increased and focused to a sufficient intensity to generate tissue destruction by heat and cavitation.

At the focal point (focused ultrasound), the high intensity waves cause an intense rise in temperature, up to 100°C, resulting in protein denaturation and coagulative necrosis. At this thermal damage, the formation of cavitations by interaction with the microbubbles in the tissue contributes to coagulative necrosis.[6] The so called “popcorn” response is the hyperechogenicity at ultrasound scan, which is the result of local cavitation and/or formation of vapor generated gaseous bubbles at the focus.

The analysis of posttreatment specimens demonstrated an intraprostatic coagulative necrosis that leads to hemorrhagic necrosis after seven days and the formation of a scar with macrophages and deposits of hemosiderin within ten weeks.[7]

Ultrasound studies and parameters for treating prostate were defined in 1992; Madersbacher in 1994 was the first who used first prototype of Sonablate 200 HIFU for benign prostatic hyperplasia and in 1995 for prostate cancer in ten patients,[8] and the first experience on organ-confined prostate cancer with the Ablatherm device was from Gelet in 1996.[9]

## HIGH-INTENSITY FOCUSED ULTRASOUND DEVICES

The first commercial HIFU machine, Ablatherm (EDAP TMS SA Vaulx-enVelin, France), was developed by EDAP and launched in Europe in 2001; the other device, called The Sonablate^®^ 500 (Focus Surgery, Inc., Indianapolis, Indiana, USA), originated at Indiana University School of Medicine in Indianapolis in the 1970s, was further developed by Focus Surgery to treat prostate cancer.

Both machines are currently used in Europe and Japan, while they are not yet approved by the US FDA.

Baseline technology of both systems is the same (each employs HIFU to generate coagulative necrosis), but there are technical differences between the two devices: Difference in imaging and therapeutic transducers, position of the patient, type of software application for treatment planning, and safety monitoring.

## ABLATHERM DEVICE

It uses two transducers, one for imaging at 7.5 MHz, other for treatment now at 3 MHz, with a unique focal point at 40 mms. The machine is complex, with an operative specific table in which patient is placed on his right side. After ultrasound-scanned reconstruction of the prostate gland, the surgeon plans the treatment on the screen, slice by slice, from bladder neck to prostate apex, and the computer automatically drives treatment according to a predefined algorithm (primary procedure, HIFU retreatment, radiation failure). For safety, the Ablatherm device includes real time ultrasound scan, active cooling of the rectal wall, stabilization of patient and rectal wall, and a continuous control of the distance between transducer and rectal wall. There has been increasing use of transurethral resection of prostate (TURP) before HIFU to reduce the size of the gland and to reduce postoperative obstructive symptoms.

## SONABLATE DEVICE

It has a single transducer for imaging and treatment at 4 MHz, but it includes two probes with different focal points: One at 40 mms from transducer, one at 30 mms from transducer.

The machine is mobile, with a main control device and a mobile cooling system. For the procedure, patient is placed at lithotomic position. After ultrasound-scanned imaging of the prostate gland, the surgeon selects the slice and the shape for treatment, from bladder neck to prostate apex. The procedure is completely surgeon-dependent, without pre-defined treatment planning. For safety, the Sonablate device includes real time ultrasound scan with visualization of pre-operative pictures, active cooling of the rectal wall, continuous and automatic monitoring of differences between pre-operative pictures and intra-operative ones. All operative procedures with Sonablate have been standardized by Illing *et al*.[10]

The HIFU treatment is generally delivered in a day-surgery setting. All patients received an enema and an antibiotical prophylaxy. Patients are anesthetized by epidural anesthesia with sedation or general anesthesia.

A foley catheter is inserted to help identify bladder neck. The HIFU probe, covered by a condom or balloon, is manually inserted into the rectum and fixed. Degassed and cooled water circulated within the rectum to cool rectal wall and to eliminate acoustic interferences between the transducer and the rectal mucosa. After selection of the treatment zone, the catheter is removed and the treatment starts. At the end of the procedure, a transurethral catheter or a percutaneous cystostomy is inserted.

Patients are discharged the next day and received antibiotics and antinflammatory drugs for at least 21 days.

The urine drainage is removed as soon as possible.

## CONTRAINDICATION

Some relative or absolute contraindications need to be excluded before an HIFU procedure.

The main and obvious contraindication is accessibility: The procedure requires a transrectal approach, and all pathologic or anatomic conditions which exclude probe introduction are absolute contraindications. All local rectal disease should be carefully excluded during the rectal examination.

Another relative contraindication is the presence of major intraprostatic calcification: The treatment requires ultrasound, and calcification acts as an acoustic barrier to ultrasound progression and diffusion. A preoperative TURP can remove them, and it can remove the other relative contraindication to HIFU which are the presence of a prominent median lobe and/or a prostate of great volume: A prostate volume >40 ml should be excluded from treatment, or two HIFU session should be considered. This is the major difficulty of HIFU, to reach the anterior parts of the prostate.

## SAFETY

Perioperative and long-term side effects following an HIFU treatment have been extensively described in many articles.

The acute urinary retention is not a perioperative complication but a normal effect induced by thermal injury and subsequent edema and swelling of the prostate, which may increase prostate volume up to 30% of its initial volume. A TURP prior to HIFU and the use of a catheter or suprapubic tube is the simplest way to solve this immediate complication.[11] Infact, combined with TURP, postoperative catheterization or prostate urethra or bladder neck stenosis sharply decrease.

Sloughing is the passage of necrotic tissue from the coagulated gland. During the period of sloughing, patient refers dysuria with irritative and/or obstructive symptoms, and eliminates debris through micturition. Symptomatic treatment with drugs is usually sufficient. An important complication, linked to necrotic tissue, is the high risk of urinary infection, which is usually managed by a long-term antibiotical prophilaxy.

Another frequent complication is bladder outlet obstruction by bladder neck and/or prostatic urethra stricture; it occurred in 3.6 to 24.5% of the cases,[12] and is usually managed with dilation. Only few cases require a TURP.

Urinary incontinence: It is usually an urge incontinence, and tends to decrease during time until the end of edema and elimination of necrotic debris. It ranges from 0.6-16%. [11] A stress urinary incontinence is rare, more frequent if apex is not clearly spared.

Impotence: The literature is controversial, but usually it occurs in the range of 20-49.8%. The preservation of the lateral edges of the prostate, the so called nerve-sparing HIFU, permits to spare erectile function as reported by Poissonier,[13] even if this decreases in erectile dysfunction rate must be balanced with a higher rate of retreatment for persistent disease.

The major complication of HIFU is uretro-rectal fistula. It usually occurs in the first two months after the procedure, and usually it occurs in patient with bladder outlet obstruction. Edema and urinary infection, together with a nonappropriate monitoring of rectal wall and a procedure in a pretreated gland (re-HIFU, HIFU for radiotherapy-relapse) are the major causes. Experience in the procedure and the addiction of cooling system and safety monitor has dramatically decreased the incidence of fistula, which now ranges between 0.5-1.2%.[14]

HIFU has no effect on metastasis. In the past, controversy remained surrounding the hypothesis that HIFU cavitation could lead to an increase of metastases. Most experimental studies have shown that high intensity ultrasound did not enhance the potential risk of metastasis, and paradoxically the study of Wu[15] showed that a substantial number of tumor patients undergoing complete HIFU may present a negative conversion of circulating tumor-specific marker as marker of circulating tumor cells, and finally, the same group[16] showed in a clinical model that HIFU induced a positive antitumor immunity through activation of population of CD4^+^ lymphocytes.

## OUTCOMES

### Primary procedure

HIFU has been applied in most of the series, and is usually recommended for patients with localized prostate cancer, cT1-cT2 N0M0 prostate cancer who are not candidates for a radical treatment. To date, data on HIFU are not yet mature to propose it as a comparable oncological alternative to radical prostatectomy or radiotherapy for patients who are otherwise suitable candidates to these consolidated radical approach, except for the advantages of high-intensity focused ultrasound, which are the possibility of an outpatient procedure, the low morbidity and no invasivity, because HIFU is repeatable and mainly because it does not preclude subsequent radical treatments.

Several publications with Ablatherm and Sonablate devices have confirmed the HIFU efficacy and safety with short and mid-term results. However, due to short follow-up (the longest follow-up reported in the literature is to date by the group of Blana which reports outcomes at a mean follow-up of 6.4±1.1 years), and different definition of end points available in the series (biochemical, disease-free survival rates with variable definitions of PSA end-points, and/or biopsy data), definitive data are not yet mature. The same problem on how interpretate oncological outcomes are for the identification of best candidates for HIFU treatment. European guidelines for prostate cancer (2008 update) do not define indication for HIFU, which is generically considered as an emerging alternative therapeutic option in the patients with clinically localized prostate cancer for low-intermediate risk patients according to D'Amico risk classification.

However, considering the characteristics of patients included in the HIFU studies, current ideal indication for HIFU as primary therapy for prostate cancer are summarized in Table 1.

**Table 1:** Ideal indication for high-intensity focused ultrasound as primary procedure

Age	>70 years
Clinical	T1-T2 N0M0 prostate cancer
Gleason score	<7
PSA level	<15 ng/ml
Prostate volume	<40 ml

The first point for best oncological outcomes is patient's selection: In most series,[17] HIFU has been applied and recommended for patients with localized prostate cancer with clinical stage T1-T2 N0M0, a Gleason score ≤7, a baseline PSA value ≤15-20 ng/ml, with a prostate volume <40 ml, who are not suitable for a radical prostatectomy or who refuse to undergo for surgery and proposed as an effective alternative to radiation therapy.[18] Some authors[19] advocated HIFU plus hormone therapy as an alternative to hormonal therapy plus EBRT in high risk prostate cancer (clinical stage up to T3a, or Gleason score 8-10 or total PSA level >20 ng/ml), reporting an interesting 77% rate of negative biopsy and good results at one year follow-up.

In Mearini series,[20] best results are achieved for patients with low risk disease [three years biochemical no evidence of disease (bNED) 86.1%; negative biopsy 75.5%] and intermediate-risk disease (bNED 79.6%; negative biopsy 77.4%) with no statistically different results, while high and very-high risk disease presented an unacceptable risk of biochemical relapse (56.4% and 19.6%, respectively; sometimes expression of systemic disease) and/or positive bioptic findings (64.3% and 81.2%, respectively).

The second point is definition of response.

An ideal result after HIFU, considered as an ablative procedure, is the achievement and maintenance of an undetectable PSA. However, several past studies have defined a complete response after HIFU as a negative control biopsy and a PSA level of less than 4.0 ng/ml, while other author[13] used progression defined as any patients with positive biopsy or a PSA more than 1 ng/ml. Other author[17] defined biochemical progression as a PSA rise over 0.4 ng/ ml, but now the most accepted definition of disease-free status is the ASTRO criteria,[21] i.e., three consecutive PSA increases after the PSA nadir have been reached. According to the ASTRO definition of bNED, Uchida *et al.*,[22] reported an overall biochemical disease-free rate of 75%, with a clear distinction for low risk disease (84%) vs. 69% and 51% for intermediate and high risk diseases, respectively. Other author used the new ASTRO-Phoenix definition of biochemical failure (i.e., PSA nadir plus 2 ng/ml): According to this definition, overall five-years bNED reached was 77%[23] in patients with low-intermediate risk prostate cancer. According to ASTRO-Phoenix criteria, in Mearini experience,[20] overall bNED is 78.2%, similar to other experience with HIFU, and the results for low risk and intermediated risk group are comparable to the outcome of patients treated with brachytherapy[24] [Table 2].

**Table 2:** Outcomes following brachytherapy or high-intensity focused ultrasound according risk classification

	Years	Low risk (%)	Intermediate risk(%)	High risk (%)
Brachytherapy				
Beyer[24]	5	88	79	65
Blasko[25]	10	94	82	65
HIFU				
Zelefsky[26]	5	88	77	38
Uchida[22]	3	92	75	64
Blana[17]	5	90	84	56
Mearini[29]	3	86	79.6	-

Response rate are defined according to PSA level, ASTRO criteria (1997), or ASTRO-Phoenix criteria (2005). Table 3 shows disease-free survival rate according to different definitions of response.

**Table 3:** Outcomes following high-intensity focused ultrasound for primary purpose

Study	Device	No. patients	Clinical stage	Definition of response	DFSR (%)	Years
Uchida[22]	S	63	T1c-2b N0M0	ASTRO 1997	75	3
Mearini[20]	S	163	T1c-T3a N0M0	ASTRO 2005	78	3
Blana[17]	A	146	T1-T2 N0M0	PSA <1 ng/ml	84	22 mo
Chaussy[11]	A	271	T1-T2 Nx/0M0	ASTRO 1997	82	3
Blana[23]	A	140	T1-T2 Nx/0M0	ASTRO 2005	59	7
Uchida[27]	S	181	T1c-T2b N0M0	ASTRO 1997	78	5
Misrai[28]	A	119	T1-T2 N0M0	ASTRO 2005	30	5
Poissonnier[13]	A	227	T1-T2 N0M0	PSA <1 ng/ml	66	5

A positive finding at prostate biopsy is an indicator of local persistence-relapse, and it is obviously another indicator of HIFU failure.

Disease control using negative biopsy findings, usually measured at 3-6 months after HIFU, in most series was observed between 66-93.4%, with difference between low/intermediate risk and high risk patients [Table 4].

**Table 4:** Prostate biopsy outcomes following high-intensity focused ultrasound for primary purpose

Study	Device	No. patients	Clinical stage	Negative biopsy, %
Uchida[22]	S	63	T1c-2b N0M0	87
Mearini[20]	S	163	T1c-T3a N0M0	66
Blana[17]	A	146	T1-T2 N0M0	93
Chaussy[11]	A	271	T1-T2 Nx/0M0	85
Blana[23]	A	140	T1-T2 Nx/0M0	86
Misrai[28]	A	119	T1-T2 N0M0	35
Uchida[29]	S	115	T1-T2 N0M0	64

Outcomes in terms of biochemical-free survival or negative biopsy obviously varied according to patients selection, i.e., baseline PSA values, stage of disease, number of positive sextant biopsies, Gleason score.

The third point is the definition of a surrogate for predicting treatment failure. Prognostic factors to estimate the risk of treatment failure would be useful for the clinician in informing patient regarding the likelihood of requiring salvage treatment. Most authors[23,29] agree that PSA nadir (i.e., the lowest postoperative PSA value, usually achieved within 3-4 months) shows a clear and intuitive association with the risk of treatment failure. PSA nadir was found to be strongly associated with preoperative baseline PSA (explained by the increasing probability of extraprostatic disease with increasing PSA level) and prostate volume (large prostate remnants will produce PSA). PSA nadir can be used to predict the risk of residual disease, showed by six months postoperative prostate biopsy. However, the correct PSA-nadir cut-off has not been yet defined [Table 5], even if, like for radical prostatectomy, a value <0.20 ng/ml seems to be the best predictor of disease persistence.

**Table 5:** Prostate-specific antigen nadir outcomes after HIFU as primary purpose

Study	Device	No. patients	Clinical stage	PSA nadir (ng/ml)
Uchida[22]	S	63	T1c-2b N0M0	0.20
Mearini[20]	S	163	T1c-T3a N0M0	0.40
Uchida[29]	S	115	T1-T2 N0M0	0.20
Blana[17]	A	146	T1-T2 N0M0	0.50
Ganzer[30]	A	103	T1-T2 N0M0	0.20
Misrai[28]	A	119	T1-T2 N0M0	1

In most contemporary series, a PSA nadir <0.5 ng/ml, a good prognostic indicator of success, is obtained in a range of 61-84%.

In Mearini experience,[20] a PSA nadir >0.40 ng/ml, correlates with biochemical relapse or positive findings at prostate biopsy, but surprisingly it did not correlate at univariate analysis with any preoperative variable (baseline PSA, prostate volume, stage, Gleason score). According to risk stratification, PSA nadir showed a linear trend, which approaches statistical significance, with an increasing value for high-very high risk disease.

### High-intensity focused ultrasound retreatment

In some cases and in the presence of a positive control biopsy, HIFU will have to be repeated. The safety profile of HIFU permits a no limited number of sessions, and up to five sessions have been described.[31]

However, the safety of a re-HIFU has been subject of discussion in the panorama of HIFU users; in particular, rate of incontinence and erectile dysfunction seems to be increased by another HIFU session, and also for urethral stenosis.

### Salvage high-intensity focused ultrasound

HIFU should be proposed as salvage after external beam radiation therapy EBRT or failure after brachytherapy, as 20-50% of the patients may experience a PSA failure over time.

Of these subject, about one-third have a true isolated local recurrence, potentially cured by a local salvage approach, even if patients with relapse after EBRT generally have a very poor prognosis, and the therapeutic options are limited.

However, before local salvage approach, true local recurrence must be documented by prostate biopsies.

Gelet in 2004,[32] Chaussy in 2006,[33] and Murat in 2007 and 2008[34,35] used HIFU as local treatment of biopsy-proven recurrence after EBRT, with a local control rate of 80%, 74%, and 73%, respectively, and with best result for patients with initial low risk or at least intermediate risk group characteristics. The use of correct indication for best results are particularly important in the setting of HIFU after EBRT or brachytherapy, because complications rate are significant, as fistula rate reaches the incidence of 7% and incontinence rate grows up to 50%, with a lot of cases of grade 3 stress incontinence.

Zacharakis, in 2008,[36] reported a retrospective evaluation of results and safety of HIFU-Sonablate in 31 patients with biopy-proven recurrence after EBRT, with a bNED of 71%. Urethral stricture rate requiring resection was reported in 36% of the cases, two patients developed grade 3 urinary incontinence and 2 a recto-urethral fistula (7%) [Table 6].

**Table 6:** Local control rate and main complications after HIFU as salvage treatment

	No. pts	Local control rate (%)	Mean follow-up (months)	G3 Incontinence (%)	Fistula (%)
Gelet[32]	71	80	14.8	7	6
Zacharakis[36]	31	93	7.4	7	6.4
Murat[34,35]	167	73	18.1	11	5

## FUTURE

The present of HIFU is to define its role in management of localized prostate cancer as primary treatment and as therapeutic option for radiation failure.

The future of HIFU will be in defining imaging modalities, the evaluation of adjuvant therapy, its use in locally advanced prostate cancer, and finally its use as a focal therapy.

HIFU is an ultrasound-guided technology, and now the presence of a color Doppler in the new software of Sonablate should help identify neurovascular bandles to spare sexual function. However, the most intriguing new is the use of HIFU MRI-guided, as a prototype of endorectal probe coupled with focused ultrasound has been develop and presented at the 2005 international society for magnetic resonance in Medicine in Miami.

New imaging methods as fat-saturated gadolinium-enhanced MRI can demonstrated accurately the extent of tissue damage induced by HIFU, and multi-sequence MRI of prostate gland should help physician to discriminate between local and systemic failure after an HIFU procedure, reducing the significance of a false-negative data of a post-operative prostate biopsy in a patients with a PSA rising.

Like for radiation therapy, the neoadjuvant or adjuvant use of hormone manipulation has not been completely evaluated, apart from an experience of Ficarra[19] who proposed a 3-year hormonal therapy with LH-RH blockade in the setting of high risk prostate cancer according to D'Amico risk classification, or the experimental findings of Paparel[37] with the use in a rat model of the synergic positive effects of HIFU and docetaxel.

The real interesting setting of HIFU will be the evaluation of its use as focal therapy in case of accurate and reliable diagnosis of monofocal prostate cancer. This involves the treatment of only the areas of prostate cancer, so sparing as much as possible the ‘health’ gland, sparing continence and potency as much as possible.

This interesting use of focal HIFU starts from the consideration that 10-40% of men with prostate cancer will have monofocal disease, and some patients with multifocal disease have only a so called ‘index lesion’ or just one monofocal clinically significative disease. The problem of focal HIFU is that monofocal prostate cancer cannot be reliably localized with current tests or intervention, and it should be overtake by a saturation biopsy and/or the use of multi-sequence magnetic resonance imaging. Previous experience with hemiablation cryotherapy by Onik[38] and Lambert[39] had shown feasibility of focal treatment prostate cancer, with interesting short terms outcomes and high rate of post-operative erectile function (86% and 71%, respectively).

The only paper on hemiablative HIFU using the Sonablate is from Muto,[40] which used HIFU as in 29 patients with biopsy-proven unilateral disease, sparing one half of the transition zone, controlateral to the disease. But his experience is too early and not conclusive.

The prospect of using HIFU as focal therapy[41] lies on accurate localization of at least significative malignant foci, and this requires a state of the art diagnostic and therapeutic imaging, and this will be the future.

## CONCLUSIONS

HIFU is a relatively new procedure of prostate cancer treatment and surely it could become one choice for patient with localized prostate cancer.

HIFU is already approved in Canada, Europe and Asia, and has gained FDA acceptance for a phase III clinical trial.

Such as others mini-invasive treatment, HIFU needs a careful selection of patients (localized prostate cancer, low risk of nodal or systemic disease, small prostate gland) and it could be reserved for patients with low-intermediate risk disease as defined by D'Amico risk stratification. ASTRO or Phoenix criteria, together with prostate biopsy and PSA nadir are the best surrogate to define disease control.

However, only more extensive follow-up study, and randomized control trial comparing HIFU with other form of treatment will definitively place HIFU in the armamentarium of prostate cancer control, because the current evaluation of oncological outcome is biased by the heterogeneity of populations in which HIFU has been used along with several definitions of treatment failure.

As the noninvasive treatment of prostate cancer, HIFU, has advantages and disadvantages, which should be considered in the decision making process [Table 7].

**Table 7:** Advantages and disadvantages of HIFU as primary treatment of prostate cancer

Advantages	Disadvantages
Outpatient procedure	Possible permanent damage to erectile function
Noninvasive	Difficult to reach the anterior parts of the gland
Low postoperative morbidity	Short follow-up
Repeatable	Difficult in definition of response
Not preclude subsequent radical treatment (surgery, radiotherapy)	
